# Evaluation of the biocompatibility of experimentally manufactured portland cement: An animal study

**DOI:** 10.4317/jced.51210

**Published:** 2014-02-01

**Authors:** Sibel Koçak, Hülya Erten, Emre Baris, Serkan Türk, Tayfun Alaçam

**Affiliations:** 1 Department of Endodontics, Faculty of Dentistry, Bülent Ecevit University, Zonguldak, Turkey; 2 Department of Restoratif Dentistry, Faculty of Dentistry, Gazi University, Ankara, Turkey; 3 Department of Oral Pathology, Faculty of Dentistry, Gazi University, Ankara, Turkey; 4 Department of Endodontics, Faculty of Dentistry, Gazi University, Ankara, Turkey; 5 Turkish Cement Manufacturers’ Association, Ankara, Turkey

## Abstract

Objectives: The purpose of this study was to evaluate the biocompatibility of MTA and the experimentally manufactured portland cement (EMPC). Study design: Twenty one Sprague Dawley (SD) rats were allocated to testing of three groups. Group I and Group II included ProRoot MTA and the EMPC. The materials were mixed with distilled water and placed in polyethylene tubes. The tubes were implanted subcutaneously in the dorsal region of the animals. Group III served as control; the implanted polyethylene tubes remained empty. At 7, 14, and 28 days after the implantation, the animals were sacrificed and the implants were removed with the surrounding tissues. The specimens were prepared for histological examination to evaluate the inflammatory response. Results: No significant difference was found between tissue reactions against the tested materials (p>0.05). Also, control group showed similar results (p>0.05). Conclusions: Results suggest that the EMPC has the potential to be used in clinical conditions in which ProRoot MTA is indicated. MTA and the EMPC show comparable biocompatibility when evaluated in vivo. Although the results are supportive for the EMPC, more studies are required before the safe clinical use of the EMPC.

** Key words:**Mineral trioxide aggregate, portland cement, subcutanous implantation.

## Introduction

Biocompatibility of a material is the ability of a material to perform with an appropriate host response in a specific situation. This means that the tissue of the patient that comes into contact with the materials does not suffer from any toxic, irritating, inflammatory, allergic, genotoxic, or carcinogenic reaction (1).

Mineral trioxide aggregate (MTA) was developed as a retrofilling material in the 1990s. A number of biocompatibility studies have been conducted either in vitro or in vivo, and the results showed that MTA presents good sealing ability and tissue healing (2-9).

The chemical, physical, and biological properties of Portland cement (PC) were analyzed. Estrela et al. (10) reported that PC contains the same principal chemical elements as MTA, except for the bismuth oxide in MTA that increases the radiopacity of the material. Saidon et al. (11) reported that MTA and PC have similar properties but that MTA is an expensive material whereas PC is an economic cement.

An experimentally manufactured Portland cement (EMPC) was developed as an alternative to MTA by the Turkish Cement Manufacturers’ Association. Components such as clay or chalk are taken directly from nature -including the arsenic- which appeared in PC. EMPC comprises pure components.

The aim of this study was to evaluate the reaction of rat subcutaneous connective tissue against the implantation of polyethylene tubes filled with MTA and EMPC.

## Material and Methods

Approval for the animal use protocol presented below was sought and given by the Animal Ethic Committees at Hacettepe University (No: B.30.2.HAC.0.01.00.05/42). 21 male Sprague-Dawley rats, each weighing 225 to 250 g, were used in this experiment. Each animal was anesthetized by an intraperitoneal injection of ketamine hydrochloride and xylazine. Afterward, the dorsal skin was shaved and disinfected.

Three incisions were made in the skin using a No. 15 scalped blade, and 2-cm pockets were created by the blunt dissection of the incisions. MTA was prepared according to the manufacturer’s instructions. EMPC powder was mixed with a sterile saline solution. Freshly mixed test materials were applied with an amalgam carrier into clean, sterile polyethylene tubes (Estern Medikit; Haryana, India) with a 1.3-mm inner diameter and 5-mm length. Each implant was carefully placed in a pocket, and the third incision received an empty sterilized tube to serve as a control. To prevent interactions of materials, the tubes were placed at least 2 cm apart. The skin was closed with 4/0 silk sutures. The evaluations were made 7, 14, and 28 days after surgical implantation. During each examination period, 7 animals were sacrificed by administration of a high dose of anesthetics. The dorsal skin was shaved, and the implants were removed with their surrounding tissues. The samples were kept in a formalin solution.

After histologic processing, the tissue was serially sectioned longitudinally with microtom (Leica SM-2000R, Leica Corp, Germany) set at 5–6 µm. The samples were stained with hematoxylin-eosin for the histological evaluation using Unna’s method for the evaluation of mast cells. Histological evaluations were made under a light microscope (Nikon Eclypse E-600, Nikon Corp, Japan) at 40 x, 100 x, 200 x, and 400 x magnification. The observer was blinded to the procedure. Evaluation of inflammatory cell and mast cell infiltration was performed according to Salman et al (12). Statistical analysis was performed using the Friedman and Wilcoxon sign tests for intragroup comparison and Kruskal-Wallis and Mann-Whitney U tests for intergroup comparison.

## Results

Macroscopic examination at the implant sites revealed that wound healing was satisfactory and without infection at all evaluation periods. The ratio of tissue reaction to the implanted materials is shown in  Table 1.

**Table 1:**
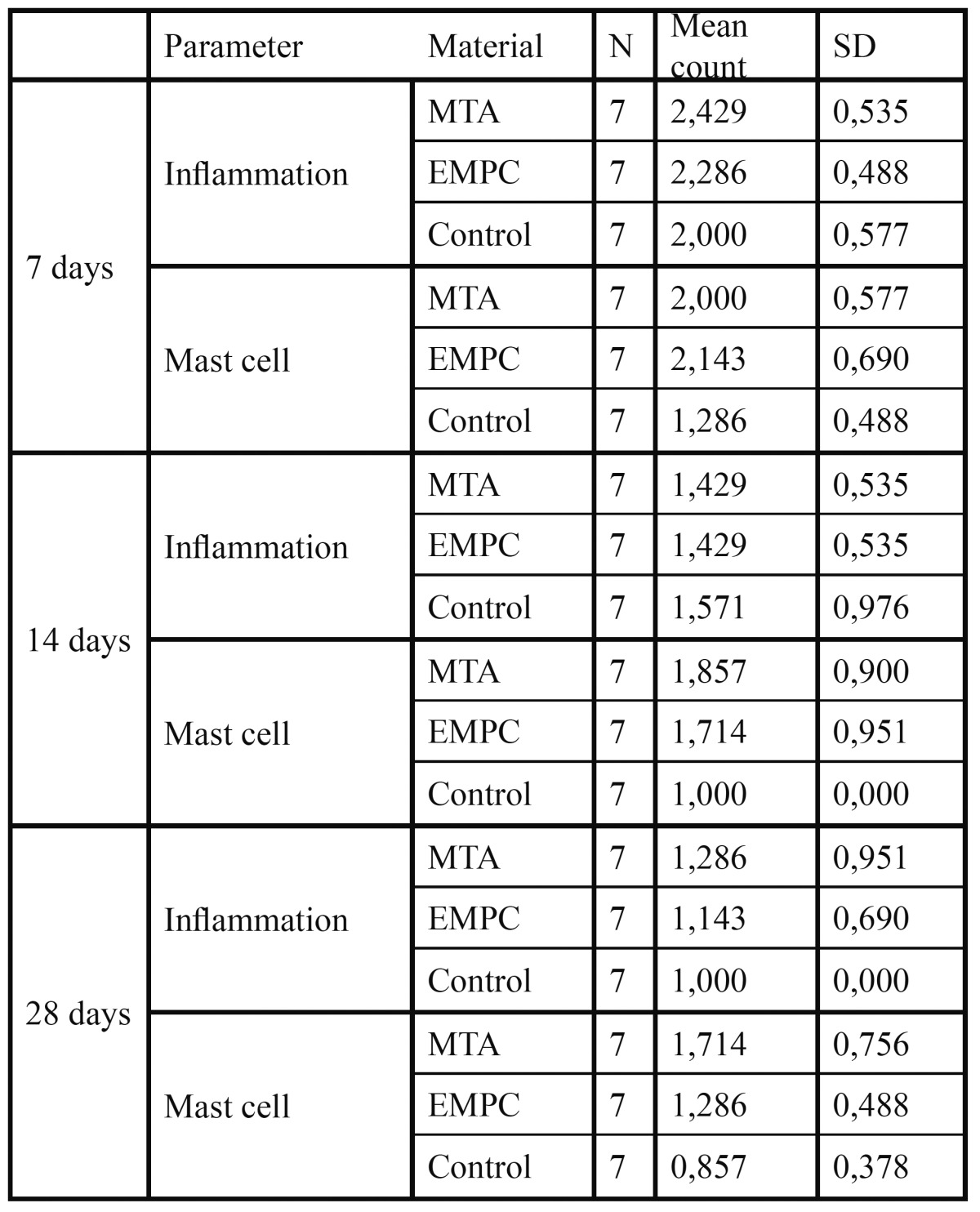
Number of implants and intensity of inflammatory response at different periods of the study

- 7 Days

**Figure 1:**
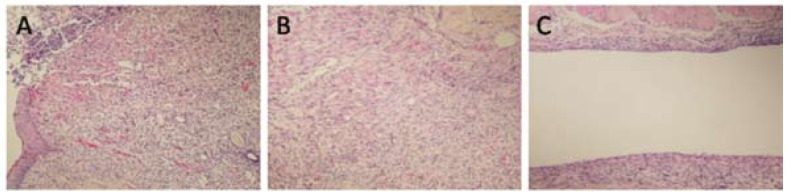
A) EMPC, 7th day (H&E, x100); B) MTA, 7th day (H&E, x100); C) Control, 7th day (H&E, x100).

The means of inflammation grades for the MTA and EMPC groups were 2.43 ± 0.54 and 2.49 ± 0.49, respectively (severe to very severe infiltration of lymphocyte and plasma cells). In the control group, the mean inflammation was 2.00 ± 0.58, which consisted of moderate infiltration of chronic inflammatory cells. There was no statistically significant difference between the groups (p<0.05), (Fig. 1).

Perivascular mast cells were observed around the end of the implants in all groups. The means of the number of mast cells for the MTA, EMPC, and control groups were 2.00 ± 0.58 2.14 ± 0.69, and 1.29 ± 0.49, respectively. There was a statistically significant difference between the groups (p<0.05); however, no difference was found between MTA and PCRA (p>0.05).

- 14 Days

**Figure 2:**
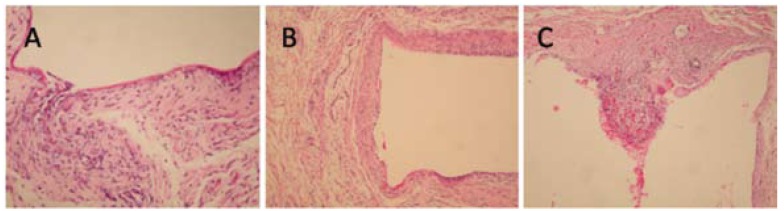
A) EMPC, 14th day (H&E, x200), B) MTA, 14th day (H&E, x100), 3c: Control, 14th day (H&E, x100).

The means of inflammation grades for both MTA and EMPC groups were 1.43 ± 0.54, which consisted of moderate infiltration of predominantly chronic inflammatory cells. In the control group, the mean inflammation was 1.58 ± 0.98, which consisted of moderate infiltration of chronic inflammatory cells. There was no statistically significant difference between the groups (p<0.05), (Fig. 2).

The means of the number of mast cells for the MTA, EMPC, and control groups were 1.86 ± 0.90, 1.71 ± 0.95, and 1.00 ± 0.00, respectively. There was no statistically significant difference between the groups (p<0.05).

- 28 Days

**Figure 3:**
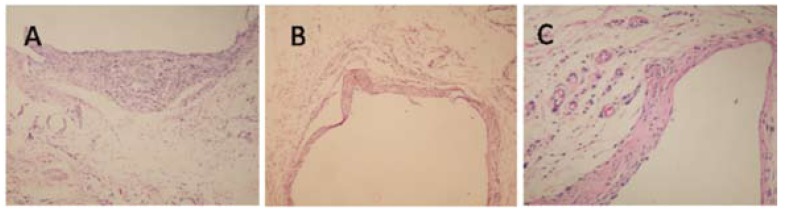
A)EMPC, 28th day (H&E, x100), B) MTA, 28th day (H&E, x100), C) Control, 28th day (H&E, x200).

The means of inflammation grades for the MTA and EMPC groups were 1.29 ± 0.95 and 1.14 ± 0.69, including mild infiltration of inflammatory cells. In the control group, the mean inflammation was 1.00 ± 0.00. There was no statistically significant difference between the groups (p<0.05), (Fig. 3).

The means of the number of mast cells for the MTA, EMPC, and control groups were 1.71 ± 0.76, 1.29 ± 0.49, and 0.86 ± 0.38, respectively. There was no statistically significant difference between the groups (p<0.05).

Mild inflammatory cell infiltration around the implants was observed in all groups after 7 and 14 days. This inflammatory infiltration comprised mostly plasma cells and lymphocytes. Numerous neutrophils were seen in the 7-day groups while the 14-day groups showed a few neutrophils. At day 28, all groups had fewer inflammatory cells and presented mature fibrous tissue.

The severity of the mast cells decreased over time; however, there was no statistically significant difference between the groups.

## Discussion

Materials must not have a deleterious effect when in contact with tissues before they are marketed and used in dental practice (13). Following ISO/6876 and 10993-5 regulations, in vitro cytotoxicity tests, such as tissue and cell culture assays, are important to provide initial evidence in the study of dental materials, and are critical to identify those components exercising cytotoxic effects (14). However, these tests lack the interaction of the material with cells in the tissue, and those attracted to the site reaction (15).

As a second step, in vivo implantation of materials in laboratory animals was proposed, which provides much more information about the inflammatory and immune responses developed by the test material (16,17). The present study evaluated the inflammatory reaction of the cellular subcutaneous tissue of rats to Teflon tubes filled with MTA or EMPC.

The subcutaneous implantation was considered a suitable secondary test for evaluation of biocompatibility properties of endodontic materials. Many studies have evaluated material biocompatibility by using different implantation vehicles, such as polyethylene tubes, silicon tubes, dentin tubes, and Teflon tubes (18-21). In the present study, Teflon tubes were used because of their inert nature and ability to bring a test material into contact with living tissue in a controlled and effective manner (22). A previous study evaluated the reaction of the subcutaneous tissue of rats after 7 and 30 days, implanted with dentine tubes filled with MTA, calcium hydroxide, or PC (23). The authors found similar results for the 3 materials in specimens stained with hematoxylin and eosin. After 7 days, a mild to moderate inflammatory reaction was observed in all groups; after 30 days, fibrous connective tissue was found in contact with the materials.

This study has demonstrated that all of groups that are implanted into the dorsal connective tissue of rats promote a moderate to severe inflammatory reaction over a 7-day period. The inflammatory reaction decreased with time. Similarly, Gomes-Filho et al. (24) showed that both MTA and Portland cement cause moderate reactions at 7 days, which decreased with time. During the 7-day control, the moderate to severe inflammatory reaction in all groups could be the result of the surgical trauma.

Connective tissue healing was remarkable for both experimental materials at the 28-day observation period. Inflammation grades at 14- and 28-day intervals for both MTA and EMPC significantly decreased when compared to 7-day controls. Similarly, Shahi et al. (25) demonstrated that Portland cement shows a statistically significant decrease in inflammation grades at 7-, 15-, 30-, and 60-day intervals. Menezes et al. (8) indicated that MTA and Portland cement show a decrease in inflammation severity in the subcutaneous connective tissue in rats at 7-, 30-, and 60-day intervals.

Tissue reactions associated with ProRoot and EMPC implants were comparable. After 28-day intervals, inflammatory processes associated with most of the implants decreased significantly, suggesting that both materials are equally biocompatible. Our results were in accordance with previously published studies in which the tissue reaction to PC was compared with the tissue reaction to ProRoot MTA (11,25,26,27).

Mast cells are key elements in the innate immune system and have been termed the antennae of the immune response for their ability to detect changes in their environment and communicate these to other cells in the vicinity. Mast cells are located throughout the body in close proximity to epithelial surfaces, near blood vessels, nerves, and glands, placing them at strategic locations to detect invading pathogens. Mast cells express a number of receptors that allow them to recognize diverse stimuli (28). In our study, we observed that EMPC stimulated mast cells much like MTA. Both materials demonstrated a similar effect on inflammatory response and wound healing.

The results of the present study demonstrate that all the implanted materials are well tolerated by tissues and have acceptable biocompatibility. However, before extrapolation of these results to an applicable human clinical situation, further studies are necessary to evaluate the suitability of the experimentally-manufactured Portland cement. In conclusion, EMPC is as biocompatible as MTA, with no significant differences.
